# A Phase III Clinical Trial of Immunogenicity and Safety of Meningococcal A and C Polysaccharide Conjugate Vaccine in Infants Aged 3–5 Months

**DOI:** 10.3390/vaccines13101023

**Published:** 2025-09-30

**Authors:** Liwei Shi, Zhe Zhang, Yuqing Liu, Maoguang Li, Weicai Lu, Yue Yang, Dan Zhao, Bin Wang, Wenjian Fang

**Affiliations:** 1 Guangxi Zhuang Autonomous Region Center for Disease Control and Prevention, Nanning 530028, China; slw0627@163.com (L.S.); luweicai91@163.com (W.L.); 2 Department of Medical Services, Beijing Zhifei Lvzhu Biopharmaceutical Co., Ltd., Beijing 100176, China; zzgzsz@163.com (Z.Z.); liuyuqing@zhifeishengwu.com (Y.L.); 3 NHC Key Laboratory of Research on Quality and Standardization of Biotech Products, State Key Laboratory of Drug Regulatory Science, National Institutes for Food and Drug Control, Beijing 102629, China; limaoguang@nifdc.org.cn (M.L.); yangyue@nifdc.org.cn (Y.Y.); zhaodan0709@nifdc.org.cn (D.Z.)

**Keywords:** meningococcal A and C polysaccharide conjugate vaccine, immunogenicity, safety, phase III clinical trial

## Abstract

**Background/Objectives:** This study aimed to evaluate the immunogenicity and safety of a Meningococcal A and C Polysaccharide Conjugate Vaccine in 3–5-month-old infants. A single-center, randomized, blinded, positive-controlled phase III clinical trial was conducted in Binyang County, Guangxi Zhuang Autonomous Region, China. Infants aged 3–5 months were randomly assigned to the experimental or control group at a 1:1 ratio. Both groups received 3 primary doses with a 1-month interval between each dose, and a booster dose administered at 18 months of age. Seroconversion rate, seropositivity rate, and GMT of bactericidal antibodies against Neisseria meningitidis groups A and C were assessed, along with adverse reactions within the full course of primary immunization and 30 days after booster immunization. **Results**: After primary immunization, in the experimental group’s pre-vaccination antibody-negative infants, seroconversion rates for groups A and C exceeded 90%, with antibody fold increases over 90 times; its seroconversion rates and GMT were non-inferior to the control group. Before the 18-month booster, the experimental group’s group A and C antibody seropositivity rates dropped to approximately 70% and 30% (slightly higher than the control), respectively. After booster immunization, the antibody levels against groups A and C were significantly higher than those before the booster and the positive rates and GMT levels of the two groups were similar. Adverse reactions were mainly solicited systemic ones (fever most common), with no statistical difference between groups. **Conclusions**: The experimental vaccine shows great immunogenicity and safety in infants aged 3–5 months and is non-inferior to the control vaccine. In the booster immunization phase of the control group, sequential vaccination with vaccines from different manufacturers was adopted, and the immunogenicity was good.

## 1. Introduction

The Gram-negative aerobic diplococcus Neisseria meningitidis (Nm) is the etiologic agent responsible for meningococcal disease. According to the different structure of the capsule, Nm has been classified into at least 12 different capsular groups, among which serogroups A, B, C, W, Y and X are the main causes of meningococcal meningitis [1,2]. Nm is the culprit behind invasive meningococcal disease (IMD), an uncommon infection characterized by rapid progression, potential lethality, and the risk of severe long-term effects in those who survive [3,4]. While IMD most commonly manifests as meningitis, septicemia, or a combination of both, it can also take other forms, including septic arthritis, pericarditis, and pneumonia [5]. IMD can lead to significant morbidity and mortality, and may also cause permanent sequelae, such as brain damage and the amputation of limbs or fingers [6,7]. In the era when the vaccine was not widely available, China had five pandemics of meningococcal disease, all of which were nationwide pandemics, occurring every 8–10 years and lasting about 3–4 years each time [8]. Nm serogroup A is the most common serogroup involved in meningococcal pandemics. However, between 2003 and 2004, the ST4821 strain of serogroup C emerged in Anhui Province, China, triggering a large-scale meningitis outbreak and this strain exhibits higher invasiveness, leading to severe complications and a higher case fatality rate compared to serogroup A [9]. Meningococcal Group A and C Polysaccharide Vaccines have been routinely administered nationwide since 2008.

IMD incidence in infants is 2–5 times higher than in children aged 1 to 4 years, with the rate being 10 times greater still when compared to individuals in older age groups [10]. Furthermore, the clinical manifestations of IMD in infants are mostly atypical. In the early stages, symptoms are often limited to ordinary fever or similar to those of non-specific viral infections, which easily leads to delayed diagnosis and treatment; moreover, the disease progresses rapidly, often resulting in death within a few hours, so vaccination for infants and young children has become a top priority [11]. As meningococcal polysaccharide antigen is a T cell-independent antigen and does not require T cell helper when stimulating B cells to produce antibodies, infants under 2 years of age have a weak immune response, only inducing a transient immune response and poor response to a booster dose [12,13]. Conjugate vaccines hold distinct advantages over polysaccharide vaccines: they are capable of triggering a protective immune response in children younger than 2 years old, offer protection that lasts for a longer period, and can enhance antibody levels when additional booster doses are administered [14].

Meningococcal A and C Polysaccharide Conjugate Vaccine produced by Beijing Zhifei Lvzhu Biopharmaceutical Co., Ltd. (Beijing, China) is a new generation of conjugate vaccine developed on the basis of the original liquid Meningococcal A and C Polysaccharide Conjugate Vaccine. The conjugate vaccine is a freeze-dried product without adjuvant and provides immune protection against group A and C meningococcal meningitis for people aged 3 months and above. The China former State Food and Drug Administration (SFDA) approved the clinical study of this product in the age-eligible population in September 2015 (Approval Letter for Drug Clinical Trial, Approval No.: 2015L02563). This study reports the results of a clinical trial in infants aged 3–5 months, who received primary immunization following the 0-, 1-, and 2-month schedule, with a booster dose administered at 18 months of age.

## 2. Materials and Methods

### 2.1. Participants and Recruitment

This study is a single-center, randomized, blinded, positive-controlled phase III clinical study. Infants aged 3–5 months are being recruited in Binyang County, Guangxi Zhuang Autonomous Region, People’s Republic of China. The eligibility criteria included the following: full-term infants (born between 37 and 42 weeks of gestation) with a birth weight ranging from 2500 g to 4000 g; axillary body temperature < 37.5 °C; written informed consent forms signed by their legal guardians; families capable of complying with the requirements of the clinical trial protocol; no history of vaccination with Meningococcal A and C Polysaccharide Conjugate Vaccine; no history of receiving other live attenuated vaccines within 14 days prior to the vaccination; and no history of receiving other inactivated vaccines within 7 days prior to the vaccination. This study was conducted after being reviewed and approved by the Ethics Committee of Guangxi Zhuang Autonomous Region (Approval Letter No.: GXIRB2020-0067; Date: 29 December 2020) and has been registered at ClinicalTrials.gov (NCT06314659). All procedures and results of the study were reported in compliance with the Consolidated Standards of Reporting Trials (CONSORT 2025).

### 2.2. Vaccines and Immunization Procedure

The study vaccine was Meningococcal A and C Polysaccharide Conjugate Vaccine (lyophilized form, 20 μg/vial, batch number: Y20200701) produced by Beijing Zhifei Lvzhu Biopharmaceutical Co., Ltd. The control vaccine in the basic immunization phase (control vaccine 1) was Meningococcal A and C Polysaccharide Conjugate Vaccine (lyophilized form, 20 μg/vial, batch number: C202008009) manufactured by Yuxi Walvax Biotechnology Co., Ltd. (Kunming, China). The control vaccine in the booster immunization phase (control vaccine 2) was Meningococcal A and C Polysaccharide Conjugate Vaccine manufactured by Chengdu Olymvax Biopharmaceuticals Inc. (Chengdu, China). All three investigational vaccines contain 10 μg each of Nm group A and group C polysaccharides (conjugated to tetanus toxoid) per 0.5 mL dose. And all are administered by intramuscular injection in the deltoid muscle on the lateral upper arm. A 3-dose primary immunization is given following the 0, 1, and 2-month schedule, and a booster dose is administered at 18 months of age.

### 2.3. Immunogenicity

All subjects underwent blood collection before the first immunization, 30 days after the primary immunization, before the booster immunization at 18 months of age, and 30 days after the booster immunization. Serum antibody assay was conducted by the National Institutes for Food and Drug Control (NIFDC). The serum bactericidal assay (SBA) was performed using rabbit complement via the triphenyltetrazolium chloride (TTC) method to measure the antibodies against Nm serogroups A and C in the subjects’ serum. Antibody levels of the subjects were evaluated at 30 days after primary immunization. The evaluation indicators included following items: (1) antibody seroconversion rate, which refers to the proportion of subjects with a pre-immunization antibody titer against Nm serogroup A or C of <1:8 who achieved a post-immunization antibody titer of ≥1:8; (2) post-immunization antibody geometric mean titer (GMT); (3) the antibody seropositivity rate (antibody titer ≥1:8 and ≥1:128). Meanwhile, the antibody levels of the subjects were investigated before booster immunization and 30 days after the booster immunization.

### 2.4. Safety

All subjects were followed up through a combination of regular follow-up visits and active reporting after each vaccine dose, with all vaccine-related adverse reactions (ARs) collected within 0–30 days post each dose. ARs occurring after vaccination were graded for severity in accordance with the Guidelines for Grading Standards of ARs in Clinical Trials of Vaccines for Preventive Use issued by China SFDA, which are categorized as Grade 1 (mild), Grade 2 (moderate), Grade 3 (severe), and Grade 4 (potentially life-threatening). All subjects were monitored for the occurrence of serious adverse events (SAE) until 6 months after the primary immunization and 1 month after the booster immunization.

### 2.5. Statistical Analysis

During the study, data collection was conducted using an Electronic Data Capture (EDC) system, with original data also preserved in paper documents. All statistical analyses were performed using Statistical Analysis System (SAS) 9.4 or later versions. When the post-vaccination seroconversion rates for both Nm serogroups A and C are expected to be 90%, it has been calculated that 252 evaluable participants per group are required. With the Bonferroni method applied for correction, this sample size enables the test to achieve a 95% power under the conditions of a one-sided significance level (α) of 0.025 and a clinical non-inferiority margin of −10%. For Serogroup A and C antibodies, post-vaccination seroconversion rates and antibody seropositivity rates were calculated for the experimental and control groups respectively. Their two-sided 95% confidence intervals (CIs) were computed via the Clopper-Pearson method, while the Miettinen-Nurminen method was used to calculate the rate difference (experimental-control group) and its two-sided 95% CI; inter-group differences were tested with the chi-square or Fisher’s exact test. For antibody GMT, data were first log-transformed, then a covariance analysis model was fitted to calculate the adjusted inter-group GMT ratio; meanwhile, log-transformed independent samples t-test was used to test inter-group GMT differences. For adverse reactions, we calculated the number of cases and incidence, and used Fisher’s exact test to compare differences between groups.

## 3. Results

### 3.1. Basic Information of Subjects

**Figure 1:**
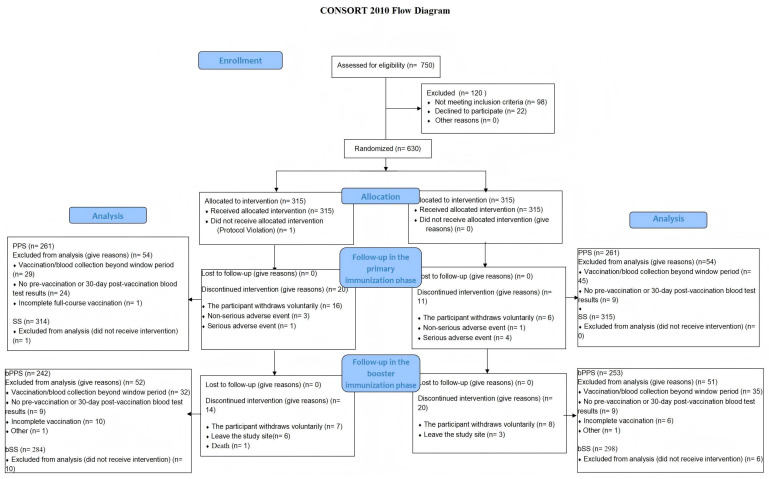
Participant disposition.

A total of 630 participants were enrolled in this trial, including 315 participants in the experimental group and 315 participants in the control group. There were 53.21% males and 46.79% females in the experimental group and 56.19% males and 43.81% females in the control group. The average age was 4.35 months in the experimental group and 4.32 months in the control group. There were no significant differences in gender or average age. See Figure 1 for details of participant screening, enrollment and completion of the clinical trial.

### 3.2. Immunogenicity Results

#### 3.2.1. Primary Immunization Phase

- **Antibody seroconversion rate in pre-vaccination antibody-negative population**

Among the pre-vaccination antibody-negative participants, the post-vaccination antibody seroconversion rates for serogroup A were 99.21% in the experimental group and 94.74% in the control group, respectively. For serogroup C, the post-vaccination antibody seroconversion rates were 91.54% in the experimental group and 89.96% in the control group, respectively. The antibody seroconversion rates for both serogroup A and serogroup C in the experimental group were non-inferior to those in the control group (with a non-inferiority margin of −10%) (Table 1).

- **Post-vaccination antibody GMT in pre-vaccination antibody-negative population**

Among pre-vaccination antibody-negative participants, the post-vaccination GMTs of serogroup A antibody were 222.5 in the experimental group and 105.8 in the control group, respectively. Compared with the pre-vaccination levels, the fold increases in their GMT values were 219.5 and 105.2, respectively. For serogroup C antibody, the post-vaccination GMTs were 91.5 in the experimental group and 70.7 in the control group, while the fold increases relative to pre-vaccination GMT levels were 91.0 and 70.1, respectively. The adjusted post-vaccination antibody GMT ratio for both serogroup A and serogroup C in the experimental group were non-inferior to those in the control group (with a non-inferiority margin of 0.67) (Table 2).

- **Rate of post-vaccination antibody titer ≥1:128 in populations with pre-vaccination antibody negativity**

In the pre-vaccination antibody-negative population, the rates of post-vaccination antibody titer ≥1:128 in the experimental group and the control group were 81.35% and 59.11% for serogroup A, and 64.62% and 53.67% for serogroup C, respectively. For both serogroup A and serogroup C, the seropositivity rates in the experimental group were significantly higher than those in the control group (*p* < 0.05) (Table 3).

#### 3.2.2. Booster Immunization Phase

Seropositivity Rate in the Total Population

Before booster immunization, the total population of the experimental and control groups showed the following serogroup A antibody titer rates: 73.14% vs. 66.01% for titers ≥1:8, and 19.83% vs. 14.23% for titers ≥1:128. At 30 days post-booster immunization, the rate of serogroup A antibody titer ≥1:8 reached 100% in both groups, while the rate of titers ≥1:128 was 96.69% in the experimental group and 96.44% in the control group. No statistically significant differences in antibody seropositivity rates were observed between the two groups for serogroup A (*p* > 0.05).

For serogroup C, before booster immunization, the rate of antibody titer ≥1:8 in the total population was 31.40% in the experimental group and 17.39% in the control group—with the experimental group showing a significantly higher rate (*p* = 0.0003). However, there was no statistically significant difference in the rate of serogroup C antibody titer ≥1:128 between the two groups (1.24% vs. 0.40%, *p* > 0.05). At 30 days post-booster immunization, the rate of serogroup C antibody titer ≥1:8 was 93.39% in the experimental group and 90.12% in the control group, and the rate of titers ≥1:128 was 61.16% vs. 52.57%, respectively; there was no statistically significant differences in antibody seropositivity rates were noted between the experimental and control groups (*p* > 0.05) (Table 4).

GMT in the Total Population

Before booster immunization, the GMT of serogroup A antibody in the total population was 18.0 for the experimental group and 11.1 for the control group, with the experimental group showing a slightly higher value (*p* = 0.0051). At 30 days after booster immunization, the serogroup A antibody GMTs were 463.2 (experimental group) and 514.8 (control group), respectively, with no statistically significant difference between the two groups. However, compared with the pre-booster period, the GMTs increased by 25.7-fold in the experimental group and 46.3-fold in the control group, and the increase multiple in the experimental group was lower than that in the control group (*p* = 0.0003).

Prior to booster immunization, the serogroup C antibody GMT in the total population was 2.9 for the experimental group and 2.2 for the control group, where the experimental group had a higher GMT (*p* = 0.0362). At 30 days post-booster immunization, the serogroup C antibody GMTs were 98.1 (experimental group) and 77.7 (control group). Compared with the pre-booster period, the GMTs increased by 34.4-fold and 35.0-fold in the two groups, respectively. No statistically significant differences were observed between the experimental group and the control group in terms of either GMT value or increase multiple (*p* > 0.05) (Table 5).

### 3.3. Safety Results

#### 3.3.1. Incidence of ARs

**Figure 2:**
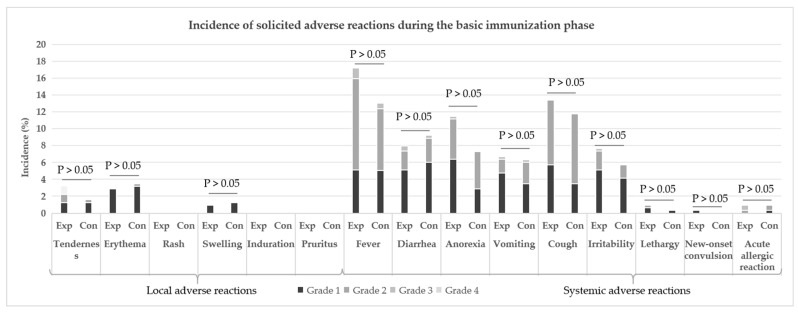
Incidence of solicited ARs during the basic immunization phase (SS). “Exp” is the abbreviation for the “experimental group”, and “Con” is the abbreviation for the “control group”.

During the primary immunization phase, ARs in both the experimental group and the control group were mainly solicited ARs, with systemic ARs being the most common. In terms of symptom analysis, fever was the predominant systemic AR, with incidence rates of 16.56% in the experimental group and 12.38% in the control group; this was followed by symptoms such as cough, anorexia, and diarrhea. For local adverse reactions, erythema was the most frequent, with incidence rates of 2.87% (experimental group) and 3.49% (control group), respectively, and tenderness and swelling were the next most common symptoms (Figure 2). There were no statistically significant differences in the incidence rates of ARs for each symptom between the two groups (*p* > 0.05).

**Figure 3:**
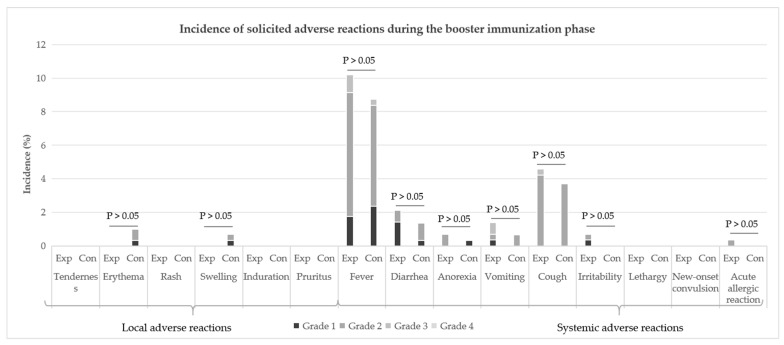
Incidence of solicited ARs during the booster immunization phase (SS). “Exp” is the abbreviation for the “experimental group”, and “Con” is the abbreviation for the “control group”.

Similar to the primary immunization phase, the booster immunization phase was dominated by solicited systemic ARs. In terms of symptom analysis, fever was the primary systemic AR in both the experimental group and the control group, with incidence rates of 10.21% and 8.72%, respectively; this was followed by symptoms such as cough, diarrhea, and vomiting. No local ARs occurred in the experimental group, while the control group had 4 cases, specifically including 2 cases of erythema, 1 case of swelling, and 1 case of erythema accompanied by swelling (Figure 3). There were no statistically significant differences in the incidence rates of ARs for each symptom between the two groups (*p* > 0.05).

#### 3.3.2. Severity of ARs

During the primary immunization phase, the incidences of grade 1, 2, 3, and 4 ARs in the experimental group were 27.71%, 26.75%, 2.23%, and 0%, respectively, while those in the control group were 22.22%, 23.17%, 1.27%, and 0.32%, respectively; the severity of ARs in both groups was mainly grade 1 and grade 2, with no statistically significant difference between the two groups (see Table 6). Specifically, 7 cases (2.23%) of grade 3 ARs occurred in the experimental group and 4 cases (1.27%) in the control group, primarily presenting as fever; no grade 4 or above ARs were observed in the experimental group, whereas 1 case of grade 4 serious AR (SAR) occurred in the control group, which was immune thrombocytopenia, and no SARs were reported in the experimental group (Figure 2 and Table 7).

During the booster immunization phase, the incidences of grade 1, 2, 3 and 4 ARs in the experimental group were 3.52%, 11.27%, 1.76% and 0%, respectively, while those in the control group were 3.36%, 12.08%, 0.34% and 0%, respectively. The severity of ARs was mainly grade 2. There was no statistical difference between the two groups (Table 7). The incidence of grade 3 and above ARs was low. There were 6 cases of grade 3 ARs in 5 patients in the experimental group, including 2 cases of fever, 2 cases of vomiting, and 1 case of fever and cough, respectively. There was 1 case of grade 3 fever in the control group. None of the patients had grade 4 and above ARs (Figure 3). No SARs occurred in either group (Table 7).

## 4. Discussion

Since the inclusion of group A meningitis polysaccharide vaccine in the routine immunization program in 1984, the cases of group A-related meningococcal meningitis have decreased significantly in China. In 2008, the inclusion of Meningococcal A and C Polysaccharide Vaccine in the Expanded Immunization Program in China further reduced the national incidence of IMD [15]. A systematic review and meta-analysis of IMD in China from 2010 to 2020 indicated that the epidemiology of IMD in China over this decade exhibited distinct serogroup distribution characteristics: among Nms strains associated with IMD, NmC (serogroup C Nm) accounted for the highest proportion (49.7%, 95%CI: 35.8–63.5%), followed by NmB (serogroup B Nm) at 30.2% (95%CI: 17.3–43.0%) and NmW (serogroup W Nm) at 23.8% (95%CI: 7.0–40.7%) [16]. Compared with people of other age groups, infants are a vulnerable population, and multiple factors collectively increase their risk of IMD—the immature immune system is a key reason for their high susceptibility to infections, while general IMD risk factors such as close contact with infected individuals, overcrowded living conditions, exposure to smoke, or concurrent viral infections also elevate their disease risk, so there is an urgent need to develop effective prevention and control strategies to protect infants from IMD [17].

A single-center, randomized, blinded, positive-controlled Phase III clinical trial was conducted in this study, targeting infants aged 3–5 months. The immunogenicity results of the primary immunization phase showed that, in the population with negative pre-immunization antibodies, both the post-immunization seroconversion rate and GMT of the experimental group were non-inferior to those of the control group. Furthermore, in this same pre-immunization antibody-negative population, the experimental group exhibited higher values than the control group in terms of the post-immunization seroconversion rate and GMT of serogroup A antibodies, and the rate of serogroup A and C antibody titer reaching ≥1:128 (*p* < 0.05). Overall, the vaccine in the experimental group demonstrated favorable immunogenicity outcomes.

Before booster immunization at 18 months of age, the rate of serogroup A and C antibody titer ≥ 1: 8 and GMT in the experimental group were higher than those in the control group, but they were significantly lower than those 30 days after primary immunization, especially when the seropositivity rate of serogroup C antibody was reduced to less than 30%, suggesting that booster immunization should be carried out timely. 30 days after booster vaccination, the antibody seropositivity rate and GMT of the whole population in the experimental group and the control group were similar. The serogroup A and serogroup C antibody levels after booster vaccination in the experimental group and control group were significantly increased compared with those before booster vaccination. The serogroup A antibody GMT was higher than the level on the 30th day after basic immunization, and the serogroup C antibody GMT was close to the level on the 30th day after basic immunization, indicating the necessity and rationality of one dose of booster vaccination at the age of 18 months for infants after basic immunization. In this trial, the control group underwent sequential vaccination with vaccines produced by different manufacturers during the booster immunization phase, and the results indicated favorable immunogenicity.

The safety results showed that during the primary immunization phase, both the experimental group and the control group were mainly affected by solicited systemic ARs. Fever was the most common among these reactions, with no statistically significant difference observed between the two groups and this is consistent with the most common ARs reported after vaccination with similar vaccines—most of these fevers were mild and transient [18], followed by other systemic reactions such as cough, anorexia, and diarrhea. For local ARs, erythema was the primary one, followed by tenderness and swelling. Regarding the incidence of all types of ARs in the experimental group and the control group, there were no statistically significant differences between the two groups. During the booster immunization phase, the most common AR in both the trial group and the control group was also fever, followed by cough, diarrhea, and vomiting and no local adverse reactions occurred in the trial group after booster immunization. In addition, the severity of adverse reactions during the primary immunization phase was mainly grade 1–2, and during the booster immunization phase was mainly grade 2. The incidence of grade 3 adverse reactions was low, and only 1 case of grade 4 SAR occurred in the control group, while no SAR occurred in the experimental group, reflecting the safety of the study vaccine.

The study results of other similar vaccines showed that the incidence of local, systemic and gastrointestinal reactions after vaccination with Meningococcal A and C Polysaccharide Conjugate Vaccine was low, mild and short-lasting, mainly redness, itching, pain, fever and vomiting [19]. Another clinical study of the similar vaccine products showed that the incidence of ARs occurring 30 days after vaccination from high to low were fever, diarrhea, injection site redness, irritability, injection site tenderness, lethargy, cough, nausea and vomiting, lactation or eating disorders, etc. [18]. The ARs in this trial were approximately the same as those of the like products, but the incidence of cough was slightly higher. Considering that the occurrence time of cough related to the study vaccine was in winter and spring, which was the season of high incidence of respiratory diseases, which affected the incidence of cough to a certain extent, and the incidence of cough was similar between the experimental group and the control group.

## 5. Conclusions

In this study, infants aged 3–5 months were given 3 doses of basic immunization according to the schedule of 0, 1, and 2 months, and 1 dose of booster immunization at 18 months of age. Results derived from this study demonstrated favorable immunogenicity and safety of the experimental vaccine. In the control group, vaccines from different manufacturers were used in the booster immunization phase, which also showed good immunogenicity.

## Figures and Tables

**Table 1:** Antibody seroconversion rate after vaccination in the population with negative antibody before vaccination during the primary immunization phase (PPS).

	Experimental Group				Control Group				Rate Difference	95%CI	*p*-Value
	*N*	*n*	%	95%CI	*N*	*n*	%	95%CI			
Serogroup A	252	250	99.21	97.16, 99.90	247	234	94.74	91.17, 97.17	4.47	1.69, 8.10	0.0035
Serogroup C	260	238	91.54	87.47, 94.62	259	233	89.96	85.64, 93.34	1.58	−3.50, 6.72	0.5352

Note: The rate difference between the two groups is calculated as (the rate of the experimental group) minus (the rate of the control group); *N* = number analyzed; *n* = number of seroconversion cases; PPS = Per Protocol Set; “%” denotes antibody seroconversion rate, calculated as (*n*/*N*) × 100%.

**Table 2:** Antibody GMT and its increase fold after vaccination in the population with negative antibody before vaccination in the primary immunization phase (PPS).

	Experimental Group			Control Group			Adjusted GMT Ratio	95%CI	*p*-Value	GMT Growth Fold		
	*N*	GMT	95%CI	*N*	GMT	95%CI				Experimental Group	Control Group	*p*-Value
Serogroup A	252	222.5	193.1, 256.4	247	105.8	87.9, 127.3	2.1	1.7, 2.6	<0.0001	219.5	105.2	<0.0001
Serogroup C	260	91.5	75.9, 110.3	259	70.7	58.1, 85.9	1.3	1.0, 1.7	0.0585	91.0	70.1	0.0579

Note: *N* = number analyzed, PPS = Per Protocol Set.

**Table 3:** Rate of antibody titer ≥ 1:128 after vaccination in negative population before vaccination in the primary immunization phase (PPS).

	Experimental Group				Control Group				Rate Difference	95%CI	*p*-Value
	*N*	*n*	%	95%CI	*N*	*n*	%	95%CI			
Serogroup A	252	205	81.35	75.98, 85.96	247	146	59.11	52.70, 65.30	22.24	14.35, 29.93	<0.0001
Serogroup C	260	168	64.62	58.47, 70.42	259	139	53.67	47.39, 59.86	10.95	2.49, 19.26	0.0112

Note: The rate difference between the two groups is calculated as (the rate of the experimental group) minus (the rate of the control group); *N* = number analyzed; *n* = Number of cases with antibody titer ≥ 1:128; PPS = Per Protocol Set; “%” denotes the rate of antibody titer ≥ 1:128, calculated as (*n*/*N*) × 100%.

**Table 4:** Seropositivity rates before and 30 days after booster immunization at 18 months of age in the whole population (bPPS).

	Antibody Titer	Before Booster Immunization			After Booster Immunization		
		Experimental Group (*N* = 242)	Control Group (*N* = 253)	*p*-Value	Experimental Group (*N* = 242)	Control Group (*N* = 253)	*p*-Value
		% (95%CI)	% (95%CI)		% (95%CI)	% (95%CI)	
Serogroup A	≥1:8 rate	73.14 (67.09–78.62)	66.01 (59.81, 71.82)	0.0849	100.00 (98.49–100.00)	100.00 (98.55, 100.00)	1.0000
	≥1:128 rate	19.83 (15.00, 25.42)	14.23 (10.17, 19.15)	0.0968	96.69 (93.59–98.56)	96.44 (93.35, 98.36)	0.8779
Serogroup C	≥1:8 rate	31.40 (25.61, 37.66)	17.39 (12.93, 22.63)	0.0003	93.39 (89.49–96.17)	90.12 (85.76, 93.50)	0.1870
	≥1:128 rate	1.24 (0.26, 3.58)	0.40 (0.01, 2.18)	0.3627	61.16 (54.70–67.33)	52.57 (46.22, 58.86)	0.0539

Note: PPS = Booster Per Protocol Set.

**Table 5:** GMT levels in the total population before booster immunization at 18 months of age and 30 days after booster immunization (bPPS).

	Before Booster Immunization			After Booster Immunization			GMT Growth Fold		
	Experimental Group (*N* = 242)	Control Group (*N* = 253)	*p*-Value	Experimental Group (*N* = 242)	Control Group (*N* = 253)	*p*-Value			
	GMT (95%CI)	GMT (95%CI)		GMT (95%CI)	GMT (95%CI)		Experimental Group	Control Group	*p*-Value
Serogroup A	18.0 (14.1, 22.9)	11.1 (8.8, 14.1)	0.0051	463.2 (412.5, 520.1)	514.8 (457.1, 579.8)	0.2109	25.7	46.3	0.0003
Serogroup C	2.9 (2.4, 3.4)	2.2 (1.9, 2.6)	0.0362	98.1 (80.9, 118.9)	77.7 (63.5, 95.2)	0.1033	34.4	35.0	0.9072

Note: PPS = Booster Per Protocol Set.

**Table 6:** Severity of ARs.

Immunization Phase	Severity	Experimental Group			Control Group			Total			*p*-Value ^1^
		Case Episodes	Case Number	% ^4^	Case Episodes	Case Number	% ^4^	Case Episodes	Case Number	% ^4^	
Primary immunization phase ^2^	Grade 1	134	87	27.71	114	70	22.22	248	157	24.96	0.1180
	Grade 2	118	84	26.75	106	73	23.17	224	157	24.96	0.3120
	Grade 3	11	7	2.23	4	4	1.27	15	11	1.75	0.3830
	Grade 4	0	0	0.00	1	1	0.32	1	1	0.16	1.0000
	Grade ≥ 3	11	7	2.23	5	5	1.59	16	12	1.91	0.5770
Booster immunization phase ^3^	Grade 1	11	10	3.52	11	10	3.36	22	20	3.44	1.0000
	Grade 2	43	32	11.27	42	36	12.08	85	68	11.68	0.7972
	Grade 3	6	5	1.76	1	1	0.34	7	6	1.03	0.1149
	Grade 4	0	0	0.00	0	0	0.00	0	0	0.00	1.0000
	Grade ≥ 3	6	5	1.76	1	1	0.34	7	6	1.03	0.1149

^1^*p*-value is calculated using Fisher’s exact probability method. ^2^ 629 cases of Safety Set (SS) were included in the basic immunization phase, 314 cases in the experimental group and 315 cases in the control group. ^3^ 582 cases of booster Safety Set (bSS) were included in the booster immunization phase, 284 cases in the experimental group and 298 cases in the control group. ^4^ “%” denotes the incidence rate of ARs, calculated as (number of cases with ARs/total number of participants in the analysis set) × 100%.

**Table 7:** Occurrence of SARs.

Immunization Phase	Adverse Event Name	Experimental Group			Control Group			Total			*p*-Value ^1^
		Case Episodes	Case Number	% ^4^	Case Episodes	Case Number	% ^4^	Case Episodes	Case Number	% ^4^	
Primary immunization phase ^2^	Serious adverse reaction	0	0	0.00	1	1	0.32	1	1	0.16	1.0000
	Blood and lymphatic system disorders	0	0	0.00	1	1	0.32	1	1	0.16	1.0000
	Immune thrombocytopenia	0	0	0.00	1	1	0.32	1	1	0.16	1.0000
Booster immunization phase ^3^	Serious adverse reaction	0	0	0.00	0	0	0.00	0	0	0.00	1.0000

^1^*p*-value is calculated using Fisher’s exact probability method. ^2^ 629 cases of SS were included in the basic immunization phase, 314 cases in the experimental group and 315 cases in the control group. ^3^ 582 cases of bSS were included in the booster immunization phase, 284 cases in the experimental group and 298 cases in the control group. ^4^ “%” denotes the incidence rate of SARs, calculated as (number of cases with SARs/total number of participants in the analysis set) × 100%.

## Data Availability

Individual participant data are available under restricted access for the requirements imposed by the Chinese Human Genetic Resources Administration concerning the public disclosure of clinical trial data. Researchers who provide a scientifically sound proposal will be allowed to access the de-identified individual participant data. Individual participant data can be obtained with a re-quest to the corresponding author (fangwenjian@zhifeishengwu.com).

## Abbreviations

The following abbreviations are used in this manuscript:

Nm	Neisseria meningitidis
IMD	Invasive meningococcal disease
SFDA	State Food and Drug Administration
NIFDC	National Institutes for Food and Drug Control
SBA	Serum bactericidal assay
TTC	Triphenyltetrazolium chloride
GMT	Geometric mean titer
ARs	Adverse reactions
SAE	Serious adverse event
EDC	Electronic Data Capture
SAS	Statistical Analysis System
95%CI	95% confidence intervals
SAR	Serious adverse reaction
PPS	Per Protocol Set
bPPS	Booster Per Protocol Set
SS	Safety Set
bSS	Booster Safety Set
NmC	Serogroup C Neisseria meningitidis
NmB	Serogroup B Neisseria meningitidis
NmW	Serogroup W Neisseria meningitidis

## References

[1] Harrison O.B., Claus H., Jiang Y., Bennett J.S., Bratcher H.B., Jolley K.A., Corton C., Care R., Poolman J.T., Zollinger W.D. (2013). Description and nomenclature of Neisseria meningitidis capsule locus. Emerg. Infect. Dis..

[2] Whittaker R., Dias J.G., Ramliden M., Ködmön C., Economopoulou A., Beer N., Pastore Celentano L., ECDC Network Members for Invasive Meningococcal Disease (2017). The epidemiology of invasive meningococcal disease in EU/EEA countries, 2004–2014. Vaccine.

[3] Aye A.M.M., Bai X., Borrow R., Bory S., Carlos J., Caugant D.A., Chiou C.S., Dai V.T.T., Dinleyici E.C., Ghimire P. (2020). Meningococcal disease surveillance in the Asia-Pacific region (2020): The global meningococcal initiative. J. Infect..

[4] Booy R., Gentile A., Nissen M., Whelan J., Abitbol V. (2019). Recent changes in the epidemiology of Neisseria meningitidis serogroup W across the world, current vaccination policy choices and possible future strategies. Hum. Vaccin. Immunother..

[5] Pace D., Pollard A.J. (2012). Meningococcal disease: Clinical presentation and sequelae. Vaccine.

[6] Brooks R., Woods C.W., Benjamin D.K., Rosenstein N.E. (2006). Increased case-fatality rate associated with outbreaks of Neisseria meningitidis infection, compared with sporadic meningococcal disease, in the United States, 1994–2002. Clin. Infect. Dis..

[7] Dinleyici E.C., Ciftci E., Somer A., Yilmaz D., Tezer H. (2025). A new quadrivalent meningococcal tetanus toxoid conjugate vaccine: Menquadfi® (MENACWY-TT). Hum. Vaccin. Immunother..

[8] Xu M., Liang Z., Xu Y., Wang J. (2015). Chinese vaccine products go global: Vaccine development and quality control. Expert Rev. Vaccines.

[9] Xu Y., Li Y., Wang S., Li M., Xu M., Ye Q. (2021). Meningococcal vaccines in China. Hum. Vaccin. Immunother..

[10] Pardo de Santayana C., Tin Tin Htar M., Findlow J., Balmer P. (2023). Epidemiology of invasive meningococcal disease worldwide from 2010–2019: A literature review. Epidemiol. Infect..

[11] Raya B.A., Sadarangani M. (2018). Meningococcal vaccination in pregnancy. Hum. Vaccin. Immunother..

[12] Harrison L.H. (2006). Prospects for vaccine prevention of meningococcal infection. Clin. Microbiol. Rev..

[13] Fukushima S., Kikuchi H., Miyazu M., Hamada A., Ouchi K., Takagi H., Mihara H., Sasaki T., Oka H., Bosch-Castells V. (2018). A Safety and Immunogenicity Study of a Single Dose of a Meningococcal (Groups A, C, W, and Y) Polysaccharide Diphtheria Toxoid Conjugate Vaccine (MEN-ACWY-D) in Healthy Japanese Participants. Jpn. J. Infect. Dis..

[14] Kim D.S., Kim M.J., Cha S.H., Kim H.M., Kim J.H., Kim K.N., Lee J.S., Choi J.Y., Castells V.B., Kim H.S. (2016). Safety and immunogenicity of a single dose of a quadrivalent meningococcal conjugate vaccine (MenACYW-D): A multicenter, blind-observer, randomized, phase III clinical trial in the Republic of Korea. Int. J. Infect. Dis..

[15] Li J., Shao Z., Liu G., Bai X., Borrow R., Chen M., Guo Q., Han Y., Li Y., Taha M.K. (2018). Meningococcal disease and control in China: Findings and updates from the Global Meningococcal Initiative (GMI). The J. Infect..

[16] Xu J., Chen Y., Yue M., Yu J., Han F., Xu L., Shao Z. (2022). Prevalence of Neisseria meningitidis serogroups in invasive meningococcal disease in China, 2010–2020: A systematic review and meta-analysis. Hum. Vaccin. Immunother..

[17] Presa J., Serra L., Weil-Olivier C., York L. (2022). Preventing invasive meningococcal disease in early infancy. Hum. Vaccin. Immunother..

[18] Wang Y.X., Wang X., Huang H.T., Zhu T., Guo W.S., Xie Z.Q., Huang L.L., Gou J.B., You W.Y., Tan J.B. (2022). Phase III clinical trial on the safety of group A+C meningococcal polysaccharide conjugate vaccine in children aged 3–23 months. Chin. J. Vaccines Immun..

[19] Fu X.S., Liang L.Y. (2011). Observation of adverse reactions after vaccination with lyophilized group A+C meningococcal conjugate vaccine. China Trop. Med..

